# RNA interference suppression of *AGAMOUS* and *SEEDSTICK* alters floral organ identity and impairs floral organ determinacy, ovule differentiation, and seed‐hair development in *Populus*

**DOI:** 10.1111/nph.15648

**Published:** 2019-01-19

**Authors:** Haiwei Lu, Amy L. Klocko, Amy M. Brunner, Cathleen Ma, Anna C. Magnuson, Glenn T. Howe, Xinmin An, Steven H. Strauss

**Affiliations:** ^1^ Department of Forest Ecosystems and Society Oregon State University Corvallis OR 97331 USA; ^2^ Department of Biology University of Colorado Colorado Springs Colorado Springs CO 80918 USA; ^3^ Department of Forest Resources and Environmental Conservation Virginia Tech Blacksburg VA 24061 USA; ^4^ National Engineering Laboratory for Tree Breeding College of Biological Sciences and Biotechnology Beijing Forestry University Beijing 100083 China

**Keywords:** *AGAMOUS*, containment, flowering, matrix attachment region, *Populus*, RNAi, *SEEDSTICK*, sterility

## Abstract

- The role of the floral homeotic gene *AGAMOUS* (*AG*) and its close homologues in development of anemophilous, unisexual catkins has not previously been studied.
- We transformed two RNA interference (RNAi) constructs, PTG and its matrix‐attachment‐region flanked version MPG, into the early‐flowering female poplar clone 6K10 (*Populus alba*) to suppress the expression of its two duplicate *AG* orthologues.
- By early 2018, six out of 22 flowering PTG events and 11 out of 12 flowering MPG events showed modified floral phenotypes in a field trial in Oregon, USA. Flowers in catkins from modified events had ‘carpel‐inside‐carpel’ phenotypes. Complete disruption of seed production was observed in seven events, and sterile anther‐like organs in 10 events. Events with strong co‐suppression of both the two *AG* and two *SEEDSTICK* (*STK*) paralogues lacked both seeds and associated seed hairs. Alterations in all of the modified floral phenotypes were stable over 4 yr of study. Trees from floral‐modified events did not differ significantly (*P *<* *0.05) from nonmodified transgenic or nontransgenic controls in biomass growth or leaf morphology.
- *AG* and *STK* genes show strong conservation of gene function during poplar catkin development and are promising targets for genetic containment of exotic or genetically engineered trees.

## Introduction

The genus *Populus* includes about 30 tree species (Eckenwalder, 1996), many of which are ecologically or commercially important. Owing to their fast growth rate, rapid transition to flowering, amenability to *in vitro* regeneration and transformation, and established reference genomes (Brunner *et al*., 2004; Tuskan *et al*., 2006; Xue *et al*., 2015; Chang *et al*., 2018), *Populus* has become an important model system for understanding both tree‐specific traits and perennial angiosperm development (Jansson & Douglas, 2007; Wullschleger *et al*., 2013).

Unlike *Arabidopsis thaliana* (*Arabidopsis*), the most thoroughly studied plant model species (Koornneef & Meinke, 2010), most *Populus* species are dioecious, and have compound, unbranched male and female inflorescences (i.e. catkins) borne separately on different trees (Eckenwalder, 1996). Depending on species and gender, each catkin can bear 20 to more than 100 flowers (Sheppard *et al*., 2000; Mohamed *et al*., 2010). Both male and female flowers in *Populus* are highly simplified and consist of only two whorls. The outer whorl is the perianth cup (i.e. cup‐shaped disk), which is thought to be developed from either fused sepals and/or petals, or an enlarged receptacle (Cronk *et al*., 2015), that supports an inner whorl of either stamens or one pistil (i.e. carpel). Guided by the *Arabidopsis*‐based ABC model of flower development (Bowman *et al*., 1991), several key floral organ identity genes have been studied in plant species with bisexual flowers (e.g. reviewed by Theißen, 2001; Causier *et al*., 2010; Litt & Kramer, 2010). However, the molecular basis for floral organ specification has not been well studied in wind‐pollinated species that have catkins with unisexual, apetalous flowers.

Much of floral development is regulated by MADS‐box genes (reviewed by Theißen *et al*., 2016). A group of well‐studied MADS genes is the *AGAMOUS* (*AG*) subfamily, which includes the eu*AG*/*PLENA* (*PLE*) lineage and the *AGAMOUS‐LIKE11* (*AGL11*) lineage (Kramer *et al*., 2004; Zahn *et al*., 2006; Dreni & Kater, 2013). The C‐function genes, *AG* in *Arabidopsis* and *PLE* in *Antirrhinum majus* (*Antirrhinum*), are key representatives of the eu*AG*/*PLE* lineage. These genes regulate the differentiation of stamens and carpels, and interact with other genes to control floral determinacy. In *Arabidopsis*, flowers in strong *ag* mutants usually lose reproductive organs and have a ‘rose‐like’ phenotype with the stamens transformed to petals and the central gynoecium transformed to a sepal‐ or petal‐looking internal structure (Bowman *et al*., 1989; Yanofsky *et al*., 1990). Similar loss‐of‐function phenotypes in the eu*AG*/*PLE* lineage have been observed in other bisexual species, such as in *Antirrhinum*, petunia, California poppy (*Eschscholzia californica*), opium poppy (*Papaver somniferum*), *Thalictrum thalictroides*, Japanese gentian (*Gentiana scabra*), *Nicotiana benthamiana*, and apple (*Malus domestica*) (Davies *et al*., 1999; Kapoor *et al*., 2002; Yellina *et al*., 2010; Hands *et al*., 2011; Fourquin & Ferrándiz, 2012; Galimba *et al*., 2012; Nakatsuka *et al*., 2015; Klocko *et al*., 2016a), and also in dioecious species such as spinach (*Spinacia oleracea* L.) (Sather *et al*., 2010). Additionally, *AG* acts as an activator of the *NOZZLE*/*SPOROCYTELESS* gene and controls pollen formation at late stages of flower development in *Arabidopsis* (Ito *et al*., 2004, 2007; Wilson & Zhang, 2009). Suppression of *AG* has been shown to cause sterility in male spinach plants (Sather *et al*., 2010).

The founding member of the *AGL11* lineage of the *AG* subfamily includes the *Arabidopsis* gene *SEEDSTICK* (*STK*; formerly known as *AGL11*) and it's orthologues (Kramer *et al*., 2004; Zahn *et al*., 2006; Dreni & Kater, 2013). *STK* has a redundant role in specifying ovule identity and also controls funiculus development and seed abscission (Pinyopich *et al*., 2003). Missense mutations or reduced expression of *STK* orthologues give rise to seedless phenotypes in cultivated grape (Ocarez & Mejía, 2016; Royo *et al*., 2018), indicating functional conservation.

RNA interference (RNAi) is a posttranscriptional‐gene‐silencing phenomenon that occurs in many eukaryotic organisms (Fire *et al*., 1998). In plant cells, it can be triggered by introducing artificial microRNA, long hairpin RNA, modified viral RNA, or synthetic small interfering RNA, and has been shown to be highly effective and stable for simultaneous suppression of multiple target genes (Small, 2007; Strauss *et al*., 2016).

Matrix attachment regions (MARs) are AT‐rich DNA elements that facilitate chromatin attachment to the nuclear matrix (Dietz‐Pfeilstetter, 2010) and have been widely used to increase the stability and level of gene expression. Results have been encouraging, but highly variable among genes, promoters, and studies (reviewed by Allen *et al*., 2000; Gelvin, 2003). However, the ability of MARs to improve RNAi suppression is almost unknown, except for a single study in haploid tobacco (*Nicotiana tabacum* cv K 326 × *N. africana*; Levin *et al*., 2005).

We report RNAi‐induced simultaneous suppression of all four *Populus* members (*AG* and *STK* orthologues) of the *AG* subfamily in the female poplar clone 6K10 (*Populus alba*), which can begin to initiate flowering within as little as 9 months after *in vitro* regeneration (Meilan *et al*., 2004). By analyzing field‐grown trees, we demonstrated that RNAi suppression resulted in stable ‘carpel‐inside‐carpel’ phenotypes, and female sterility. The presence of flanking MARs in the RNAi construct enhanced suppression efficiency three‐fold. None of the floral‐modified events showed detectable changes in tree growth or leaf morphology. Our results indicate that the *AG* and *STK* genes are functionally conserved in *Populus* and that *AG/STK* impairment is a powerful tool for genetic containment when needed for mitigation of gene flow (Daniell, 2002; Brunner *et al*., 2007).

## Materials and Methods

### Plasmid construction

**Figure 1:**
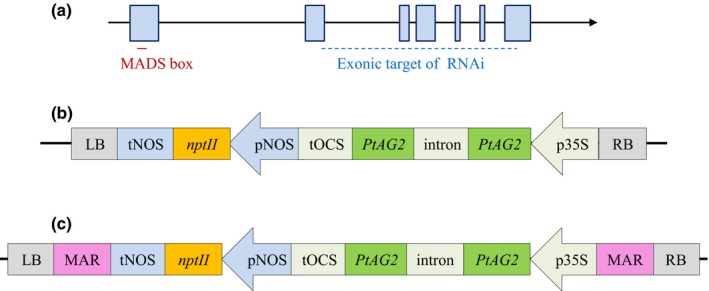
The PtAG2‐derived RNAi hairpin target and construct design. (a) Transcript of the *PtAG2* gene. Exons are indicated by boxes. The portion of the complementary DNA sequence used to make the RNA interference (RNAi) hairpin is indicated by a dashed blue line. The location of the MADS box is indicated by a red line. (b)  Diagram of the RNAi construct PTG. (c) Diagram of the RNAi construct MPG. LB, left border; tNOS, terminator of the nopaline synthase gene; *nptII*, the neomycin phosphotransferase gene; pNOS, nopaline synthase promoter; tOCS, terminator of the octopine synthase gene; *PtAG*‐intron‐*PtAG*, inverted repeat of a segment of the *PtAG2* gene; p35S, cauliflower mosaic virus 35S promoter; RB, right border; MAR, matrix attachment region derived from the tobacco *RB7* gene. Arrows indicate the direction of transcription.

We first created an RNAi hairpin by cloning two copies of *Populus trichocarpa AG2* (*PtAG2*, Potri.011G075800, *P. trichocarpa* v.3.1, phytozome v.12) complementary DNA (cDNA) fragment into the gene‐silencing vector pHannibal (Wesley *et al*., 2001). The *PtAG2* fragment is 386 bp long, consists of nucleotides 458–843 of the *PtAG2* cDNA, and contains partial or full sequences of the second to the seventh exons of *PtAG2* (Fig. 1a). We inserted two inverted copies of this *PtAG2* cDNA fragment, separated by the pHannibal intron, between the cauliflower mosaic virus 35S promoter (p35S) and the octopine synthase terminato (tOCS) in the pHannibal vector. To create the PTG construct (Fig. 1b), we then cloned the RNAi transgene (p35S:*PtAG2*:intron:*PtAG2*:tOCS) into the binary vector pART27 (Gleave, 1992). To create the MPG construct (Fig. 1c), we first inserted the RNAi transgene into the *Not1* site of pG3KM (Li *et al*., 2008a). We then excised the region between the transfer DNA (T‐DNA) borders with *Acs1* and inserted it into a modified pART27 vector in which the T‐DNA region between the *Not1* sites was removed and replaced with an *Acs1* linker.

### Amplification and sequencing of *PaAG* cDNA from the 6K10 clone

We used primers specific to *PtAG1* (Potri.004G064300) and *PtAG2* (Potri.011G075800) (Supporting Information Table S1) to amplify transcripts of *PaAG1* and *PaAG2* from the female poplar clone 6K10 (*Populus alba*). Transcripts were amplified from floral cDNA isolated from field‐grown trees. The PCR amplicons were sequenced using Sanger Sequencing at the Center for Genome Research and Biocomputing at Oregon State University (OSU), USA. Pairwise percentage sequence identities between the *PaAG* transcripts and the RNAi hairpin were calculated using clustal omega (Goujon *et al*., 2010; Sievers *et al*., 2011).

### Transgenic plants

The PTG and MPG constructs were transformed into *Agrobacterium tumefaciens* strain ‘AGL1’ using a freeze–thaw method, and then transformed into the 6K10 clone using published methods (Confalonieri *et al*., 2000). Kanamycin was used to select for transgenic tissue, and timentin was used to select against *A. tumefaciens* during plant transformation and regeneration. Presence of the intact RNAi transgene was confirmed by PCR amplification of two fragments. First, we amplified a 700 bp fragment encompassing part of *p35S* and the inverted repeat (i.e. *PtAG2:intron:PtAG2*). Second, we amplified a 600 bp fragment covering part of *tOCS* and the inverted repeat. Transgenic plants that were verified by PCR (i.e. transgenic events) were transplanted into a clone bank with nontransformed (NT) 6K10 plants. Vegetative cuttings from transgenic events and NT controls were rooted by Broadacres Nursery (Hubbard, OR, USA), and transplanted to small pots in a glasshouse at OSU before being transplanted into the field.

### Field site establishment and management

A total of 35 transgenic events were planted in Corvallis, Oregon, in late June 2011. This planting included 24 NT control trees plus 22 PTG‐transformed events and 13 MPG‐transformed events (one to eight ramets each). The overall field trial, which consisted of three poplar clones and 3477 trees transformed with 23 constructs, was established as a randomized split‐block design with two blocks per clone (Klocko *et al*., 2016b, 2018). Within each 6K10 block, six pairs of NT control trees and one to four ramets of each transgenic event were planted. In most cases, two ramets of one event were planted adjacent to one another within each block. The field was drip irrigated every night from June to September in 2011 and 2012. Weeds were controlled by hand pulling and mowing. Tree survival was recorded twice a year during winter and spring.

### Floral bud flush and floral morphology

The stage of floral bud development was measured from late January to early April using the scoring system described by Klocko *et al*. (2016b). The trees were measured twice a week in 2015 and 2016, and every 2 wk in 2017. Floral buds and catkins were photographed in the field using a digital camera (Canon Rebel, XSi; Canon, Tokyo, Japan). From 2015 to 2017, developing and mature catkins were collected from each flowering tree from late February to early April. The catkins were hand dissected and photographed using a digital microscope (Keyence VHX‐1000; Keyence America, Elmwood Park, NJ, USA), and the images were used to examine floral morphology and the presence of ovules or seeds.

### Seed germination

Two to six mature catkins (with individual flowers developed into seed pods) were collected from the PTG and MPG events and NT control trees in early April 2016 and stored dry at room temperature for 1 wk before the germination test. Seeds that were released naturally or manually removed from dried seed pods were plated in Petri dishes containing water–agar medium (1% agar, w/v). The Petri dishes were sealed with parafilm, kept on the benchtop, and usually germinated in 2 d. The number of seeds plated and germinated were recorded immediately after plating and 4–6 d later (i.e. after all viable seeds had germinated).

### Identification of potential off‐target genes

To identify potential off‐target genes, the 386 bp *PtAG2* cDNA sequence (for making RNAi hairpin) and several of its 100 bp fragments were used as blastn (default settings with an expected threshold of 10) queries to the *P. trichocarpa* genome (v.3.1; phytozome v.12). For each blastn hit, its cDNA sequence was aligned against the *PtAG2* cDNA sequence using clustalW (Larkin *et al*., 2007), and its expression in female floral tissue was calculated by averaging fragments per kilobase of transcript per million mapped reads values from three *Populus*‐based gene expression experiments (BESC423.ZL female early, BESC443.ZG female receptive, and BESC842.ZI female late, available on phytozome v.12). We used two parameters to choose genes for expression analysis: the maximum length of contiguous nucleotides that were identical to the 386 bp *PtAG2*‐cDNA sequence, and the level of expression in female flower.

### Gene expression analysis with quantitative RT‐PCR

Total RNA was isolated from developing floral buds (collected on 7 December 2016) using a cetyltrimethylammonium bromide based RNA extraction method (Gambino *et al*., 2008). RNA was treated with DNase (DNase I, amplification grade; Invitrogen), and then used for cDNA synthesis using SuperScript III Reverse Transcriptase (Invitrogen), according to the manufacturer's protocols. Quantitative RT‐PCR (qRT‐PCR) was performed using a StepOnePlus real‐time PCR system (Applied Biosystems, Foster City, CA, USA) and Platinum SYBR Green qPCR SuperMix with ROX reference dye (Invitrogen). The *ELONGATION FACTOR1‐BETA* gene (*EF1‐beta*, Potri.009G01860) and the *EUKARYOTIC TRANSLATION INITIATION FACTOR 5A* gene (*eIF5A*, Potri.018G10730) were used as reference genes. All gene‐specific primers were designed using primer3plus(Untergasser *et al*., 2012), apart from those for amplifying *EF1‐beta* and *eIF5A*, which were adopted from Wang *et al*. (2016). The efficiency of each primer pair was evaluated with standard curve before performing qRT‐PCR (Table S1). Two biological replicates (individual ramets from separate blocks, or pools of two adjacent ramets from a two‐tree row plot each in a single block) were used for each event and NT control; three technical replicates were used for each reaction. The PCR program consisted of an initial denaturation at 95°C for 10 min, 40 cycles of 95°C for 15 s and 60°C for 1 min, followed by melt‐curve analysis with a temperature increase of 0.3°C s^−1^. The relative gene expression was determined using the ΔΔ*C*_t_ method.

### Vegetative data collection

Three growth traits were measured: tree height, trunk diameter at knee height (45 cm above the ground), and trunk diameter at breast height (137 cm above the ground). We also measured five leaf morphology traits: leaf area, leaf dry weight, Chl content, petiole length, and petiole width. Growth traits were measured in January and leaf traits were measured in July of 2015, using the methods reported by Klocko *et al*. (2016b). Two derived traits, trunk volume index (= the volume of a basal frustum + the volume of an upper cone; Klocko *et al*., 2016b) and leaf density (= dry leaf weight/leaf area, also called specific leaf weight), were calculated to evaluate growth and leaf morphology.

### Statistical analysis

We used ANOVA and Tukey's honestly significant difference to evaluate differences in gene expression between the RNAi transgenic events and NT control. Using the same approach, we also examined differences among constructs and floral morphologies. Because all NT control events flowered normally, we had five category combinations of construct (PTG, MPG, and NT) and floral morphology (normal and altered): PTG/Altered (PTG/A), PTG/Normal (PTG/N), MPG/Altered (MPT/A), MPG/Normal (MPG/N) and NT/Normal (NT/N). Pearson correlation coefficients were calculated based on event mean to examine the correlation in expression between different genes. For *PaAG*s and *PaSTK*s, we calculated and compared their relative suppression levels. We first subtracted the mean expression level in each event from the mean expression level in NT control and then divided the difference in means by the mean expression level in the NT control. We then performed a paired *t*‐test on percentage suppression per event for each paralogous gene to examine the difference in the suppression.

We used a linear mixed effects model to test whether the RNAi construct and resulting flowering phenotypes affected tree growth or leaf traits. The linear mixed effect model included the fixed effect of category (five category combinations of construct and floral morphology described earlier), the fixed effect of block, the random effect of event‐within‐category, and the residual error. Assumptions of homogeneous variance and normality of errors were checked graphically with residual plots. When the assumption of homogeneous variance was violated, variances were allowed to differ by category.

For leaf traits, we performed the analysis on original measurements. For growth traits, we first performed spatial adjustment on original data using the methods reported by Klocko *et al*. (2016b). We previously compared analyses of spatially adjusted vs nonadjusted growth traits, and based on the Akaike information criterion we found the analyses of spatially adjusted data yielded a slightly better model fit (lower Akaike information criterion; Klocko *et al*., 2016b). Thus, we used the same approach in this study. Briefly, the adjusted data consisted of residuals from a model that accounted for three poplar clones transformed with 23 different constructs, macroenvironmental variation among clonal blocks, and microenvironmental variation specified by tree position (i.e. row and column). The final adjusted data points were calculated by adding the predicted mean of the trees analyzed (all PTG, MPG and NT 6K10 trees of interest) to each individual model residuals. The adjusted data were then analyzed using the linear mixed effects model described earlier, with the fixed effect of block removed from the models (block effects had been accounted for in the spatial adjustment).

Model fitting was performed using the package nlme (Pinheiro *et al*., 2018) and estimated marginal means and 95% confidence limits from the fitted models were obtained using the package emmeans (https://CRAN.R-project.org/package=emmeans). Apart from spatial adjustment, which was performed using Sas, all statistical analyses were performed in R v.3.5.0 (R Core Team, 2018).

## Results

### The *PaAG* transcripts shared high sequence identity with the *PtAG2* fragment used for making the RNAi constructs

To evaluate sequence similarity among *AG* homologues, we amplified 978 bp *PaAG1* cDNA and 887 bp *PaAG2* cDNA, covering the partial 5′ untranslated region, the full coding region, and the partial 3′ untranslated region, from the 6K10 clone using gene‐specific primers (Table S1). Analysis of the sequenced transcripts showed that *PaAG1* (GenBank accession no. MK287862) and *PaAG2* (GenBank accession number MK287861) were very similar to each other (Fig. S1), with a sequence identity of 88.83%. Additionally, *PaAG1* and *PaAG2* showed 90.67% and 98.19% sequence identity, respectively, to the *PtAG2* fragment used in the RNAi constructs. The high similarity among these *AG* cDNA sequences suggested a high probability of simultaneous suppression of the two *PaAG* genes using one hairpin RNA derived from the *PtAG2* cDNA.

### RNAi transformation produced events with modified floral morphology

As of early 2018, all trees of interest, including 22 PTG events, 13 MPG events, and 24 NT controls, had survived in the field. All events, apart from event MPG 91‐2, had at least one ramet that flowered, and 23 out of 24 NT control trees had flowered (Tables 1, S2. S3). Among these flowering events, six out of 22 (27.3%) PTG events and 11 out of 12 (91.7%) MPG events showed modified floral morphology.

**Table 1:** Event survival, flowering and floral morphology of *Populus alba* in 2018

Construct	No. of events planted in 2011	Survival, no. (%)	Flowering, no. (%)	Altered floral morphology, no. (%)	Infertile, no. (%)
PTG	22	22 (100.0)	22 (100.0)	6 (27.3)	3 (13.6)
MPG	13	13 (100.0)	12 (92.3)	11 (91.7)	7 (63.6)
NT	24	24 (100.0)	23 (95.8)	0 (0.0)	0 (0.0)

PTG, RNA interference (RNAi) construct without matrix attachment regions (MARs); MPG, RNAi construct with flanking MARs; NT, nontransgenic control. Data indicate that at least one ramet of the event has survived, flowered, showed an altered floral morphology (i.e. ‘carpel‐inside‐carpel’ phenotype or anther‐like organs) or was infertile.

**Figure 2:**
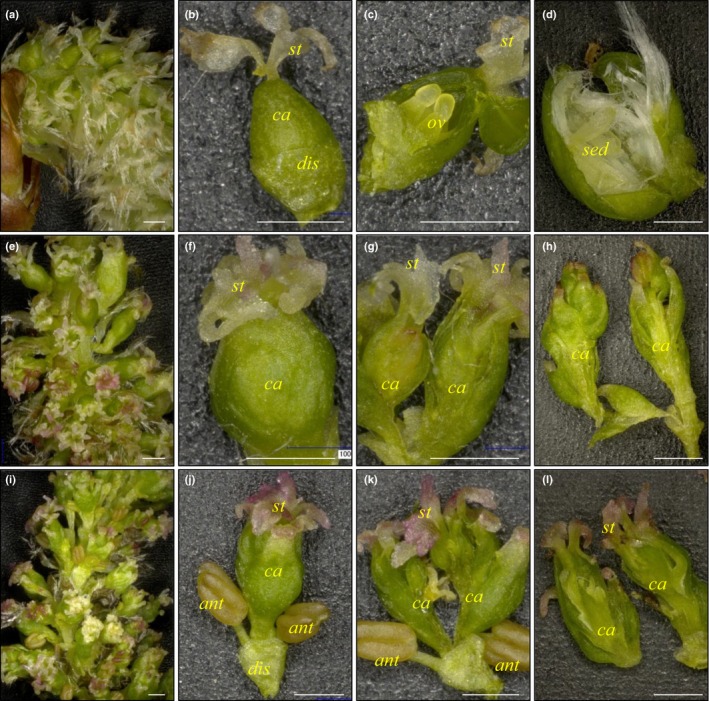
Layered carpels and anther‐like organs observed in altered events. (a) A nontransformed (NT) control *Populus alba* catkin. (b) An individual flower from the NT control catkin. (c) Hand‐sectioned NT control flower with ovules. (d) Hand‐sectioned NT control flower with immature seeds and cottony seed hair. (e–h) Catkin and flowers from altered event PTG 199 (e–g) or PTG 6‐2 (h). (i–l) Catkin and flowers from altered event MPG 165‐1 (i–k) or MPG 211 (l). *st*, stigma; *ca*, carpel; *dis*, cup‐shaped disk; *ov*, ovule; *sed*, seed; *ant*, anther‐like organ. Bars, 1 mm. Images a–c, e–g and i–k taken on 20 February 2015; images d, h and l taken on 21 March 2016.

**Figure 3:**
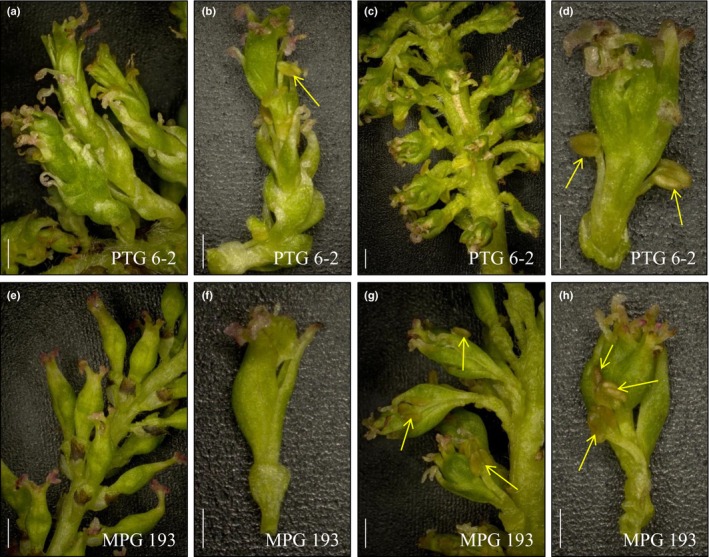
Morphological variation was commonly observed within events. (a–d) *Populus alba* catkins collected from the same branch from event PTG 6‐2 (a, c) and individual flowers isolated from these catkins (b, d). (e–h) Catkins collected from the same branch from event MPG 193 (e, g) and individual flowers isolated from these catkins (f, h). Bars, 1 mm. Arrows in images b, d, g and h indicate anther‐like organs. All images taken on 21 March 2016.

Catkins from the NT controls often carried about 50–80 female flowers, which had four stigmas and several ovules (Fig. 2a–c). White seed hairs could be observed upon flower maturation and seed development (Fig. 2d). Although catkins on the altered PTG and MPG events had about the same numbers of flowers organized in a similar way to NT controls (Fig. 2e,i), flowers from these events often had more than four stigmas and showed a ‘carpel‐inside‐carpel’ (or layered) phenotype (Fig. 2e–l). Ten out of the 17 altered events had flowers with anther‐like organs (Tables S2, S3). Some anthers appeared to be well developed in shape and were attached to the flower via filaments (Fig. 2i,k), whereas others did not have the supportive filaments and fused together with the carpel tissue (e.g. Fig. S2e,f). No pollen grains were observed in any of these anthers. Dissected flowers from several altered events had no ovules or seeds visible (Fig. 2g,h,k,l). Variation in the number of carpel layers and the presence and the appearance of anthers were prevalent both among (Fig. S2) and within events (Fig. 3). For example, catkins collected from one branch of PTG event 6‐2 differed in the numbers of carpel layers, which ranged from two to seven (Fig. 3a–d). Similarly, flowers from different catkins collected from the same branch of MPG event 193 had either no anthers or several anthers (Fig. 3e–h). Moreover, anthers from the same flower differed in their structures (i.e. with or without filament and whether or not fused to carpel; Fig. 3h). Despite these variations in the RNAi‐resulting phenotype, the degree of floral modification was stable within events during the entire study period (Fig. S3).

Besides alteration in floral morphology, we observed that floral buds on altered events flushed earlier than those on normal events and NT control trees. In 2015, 14.3% altered PTG events and 32.1% altered MPG events had already flushed by late January when we started our observations, and none of the normal events or NT controls trees had initiated flushing at that time (Fig. S4a). In 2016, 56.3% of altered PTG events and 50.0% of altered MPG events had broken their buds at the beginning of February, whereas buds on normal events and NT control trees did not flush until a week later (Fig. S4b). Similar trends were observed in 2017 (Fig. S4c–e). We also noticed that catkins on altered events often started to senesce and turn yellow and/or brown earlier than those on NT and normal RNAi events (e.g. Fig. S3a,b,e,f).

### Sterile catkins were produced from both constructs

**Figure 4:**
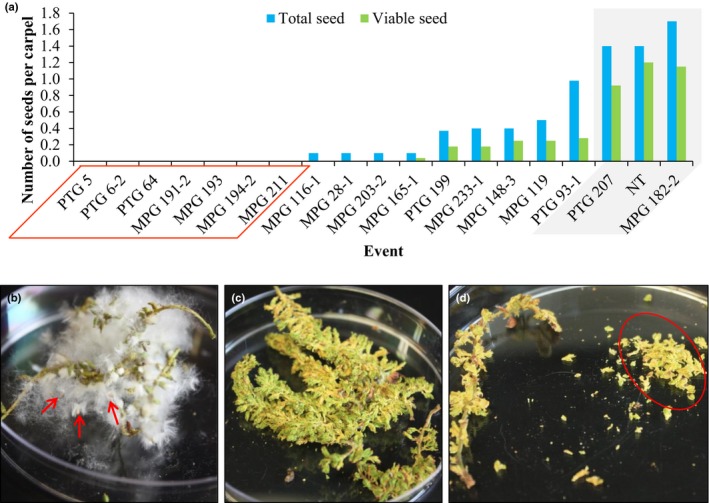
Both constructs produced events that lacked seeds and seed hair. (a) *Populus alba* seed production and germination by event. Normal events and nontransformed (NT) control are highlighted in gray. Seedless events are indicated by the orange box. (b) Seed pods from NT control released seeds (indicated by red arrows) and cottony seed hair upon maturation and drying. (c) No seed hair was produced or released from dry seed pods from altered event PTG 6‐2. (d) No seed hair was detected in hand‐dissected seed pods (indicated by red circle) from altered event MPG 211. Images (b–d) taken on 22 April 2016.

To examine seed viability, we collected mature catkins from five altered PTG events, 11 altered MPG events, two normal events (one from each construct), and two NT control trees in early April 2016. At this time, individual seed pods had just opened and seeds were about to be released. One altered event, PTG 11, had only one ramet that was flowering, and it had very few catkins at the top of its crown, which were beyond our reach; we were therefore unable to get catkins from this event. For each collected event and the NT control, we dissected 15–97 individual seed pods to check for seed production (Table S4). We found that normal catkins (from NT trees and normal events) produced a total of 1.40–1.67 seeds per seed pod, and 0.92–1.20 viable seeds per seed pod (Fig. 4a; Table S4). Although six altered events (PTG events 199 and 93‐1 and MPG events 165‐1, 233‐1, 148‐3, and 119) maintained some fertility, they showed a reduction in both total seed production (ranging from 0.10 to 0.98 total seeds per seed pod) and viable seed production (ranging from 0.04 to 0.28 viable seeds per seed pod). Seven altered events (PTG events 5, 6‐2, and 64 and MPG events 191‐2, 193, 194‐2 and 211) produced no seeds at all. Unlike normal events that released cottony seed hair upon maturation (Fig. 4b), these seedless events also produced no seed hair in their seed pods (Fig. 4c,d). Three altered events (MPG events 116‐1, 28‐1 and 203‐2) produced seeds with a rate lower than 0.98 seeds per seed pod, but none of the seeds were viable. Taken together, 10 out of the 16 altered events examined were infertile.

### Reduced *PaAG* expression was found in all altered events

**Figure 5:**
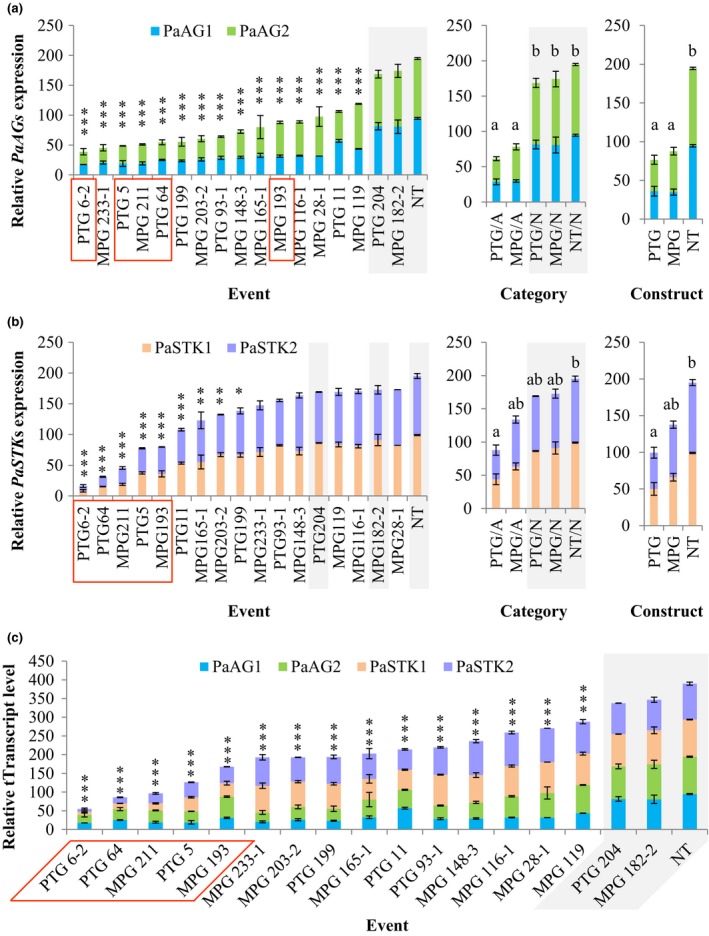
Relative transcript level of *PaAGs* and *PaSTKs* in developing floral buds. (a) Relative *PaAG*s expression by event, category (combinations of construct and floral morphology), and construct. (b) Relative *PaSTK*s expression by event, category, and construct. (c) Combined *PaAGs* and *PaSTKs* expression by event. Error bars represent ± SE of means. ***, *P* < 0.001; **, *P* < 0.01; *, *P* < 0.05, compared with nontransformed (NT) control. Different letters denote significant differences (*P *<* *0.05) in total expression of both orthologues. Normal events and NT control are highlighted in gray. Seedless events are indicated by orange boxes. PTG/A, altered PTG events; PTG/N, normal PTG events; MPG/A, altered MPG events; MPG/N, normal MPG events; NT/N, nontransgenic control.

We performed qRT‐PCR to determine the relative expression levels of the two *PaAG* genes in floral buds compared with two reference genes, *EF1‐beta* and *eIF‐5A*. Because we were unable to obtain floral buds from two altered MPG events, we examined 15 out of the 17 altered events together with two normal events and two NT control trees. As predicted, all altered events had significantly reduced expression in both *PaAG1* and *PaAG2*, ranging from 17.37% to 57.00% for *PaAG1* and 21.43% to 75.38% for *PaAG2*, compared with NT control (*P *<* *0.001; Fig. 5a and Table S5). Both constructs, PTG and MPG, conferred similar levels of suppression; they gave nearly a three‐fold decrease in the total expression of both *PaAG* genes in their altered events. Neither of the normal PTG event or MPG event showed significantly reduced expression (*P *<* *0.05) in *PaAG1* or *PaAG2*. The suppression of the two *PaAG* genes was highly and significantly correlated (*P *<* *0.001), with a Pearson correlation coefficient of 0.90 (Table S6). Additionally, on average, the suppression level of *PaAG1* in RNAi events was statistically significantly (10.6%; *P *<* *0.01) higher than that of *PaAG2*.

### Two *PaSTK* genes were found to be suppressed in altered events that failed to produce any seeds

To evaluate if there were any other genes being suppressed by the RNAi constructs, we used full‐length and fragments of the 386 bp *PtAG2* cDNA cloned into the RNAi construct as queries and performed blastn against the *P. trichocarpa* genome (v.3.1). We identified a total of 13 potential off‐target genes that had four to 14 contiguous nucleotides identical to the *PtAG2* cDNA fragment present in the RNAi constructs (Table S7). Five of these genes (*Potri.013G104900*/*PtSTK2*,* Potri.019G077200*/*PtSTK1*,* Potri.006G031600*,* Potri.001G254300* and *Potri.013G043100*), had at least one set of eight or longer contiguous nucleotides that was identical to the *PtAG2* cDNA fragment. Some of these genes (e.g. *Potri.006G031600* and *Potri.010G027000*) had no or very low expression in flowers; thus, we excluded these genes from gene expression analysis. We selected *Potri.013G104900* (*PtSTK1*) and *Potri.019G077200* (*PtSTK2*) for qRT‐PCR analysis because they are MADS‐box genes and share highest sequence identity with the *PtAG2*‐derived RNAi hairpin, and therefore are likely to be targeted by the RNAi constructs. We also examined *Potri.001G254300*, a non‐MADS‐box gene moderately expressed in flowers. However, we did not expect to observe reduced expression in this gene because it has only one set of eight contiguous nucleotides identical to the hairpin RNA.

As predicted, we observed significantly reduced expression (*P *<* *0.05) in the two *PaSTK* genes (Fig. 5b and Table S5) but not in *Potri.001G254300* (Fig. S5 and Table S5) in examined events. The expression of *PaSTK‐1* was significantly suppressed in 10 out of the 15 altered events examined, ranging from 8.37% to 71.55% in these events relative to NT control. Significant suppression of *PaSTK‐2* was observed in eight altered events, with a relative expression level of 7.93–67.70% compared with NT control. The combined expression of the two *PaSTK* genes was significantly reduced in nine altered events. Interestingly, we noticed that PTG events 5, 6‐2 and 64 and MPG events 193 and 211, which had more than 50% reduction in both *PaSTK* genes and the lowest combined *PaSTK* expression, were those previously identified to be seedless (discussed in the Sterile catkins were produced from both constructs section; Fig. 4). These seedless events also had the lowest combined expression of *PaAG*s and *PaSTK* s (Fig. 5c). The correlation between *PaSTK‐1* and *PaSTK‐2* expression was very high, with a correlation of 0.97 (*P *<* *0.001); the correlations between *PaAG*s and *PaSTK*s expression were lower than 0.68 (*P *<* *0.01 for *PaAG1* and *PaSTK‐1*, and *PaAG2* and *PaSTK*s; *P *=* *0.04 for *PaAG1* and *PaSTK‐2*; Table S6). Also, we found that *PaSTK1* showed 5.3% higher mean suppression than *PaSTK2* did in the RNAi events (*P *<* *0.01).

### Transformed poplar trees had normal vegetative growth

**Figure 6:**
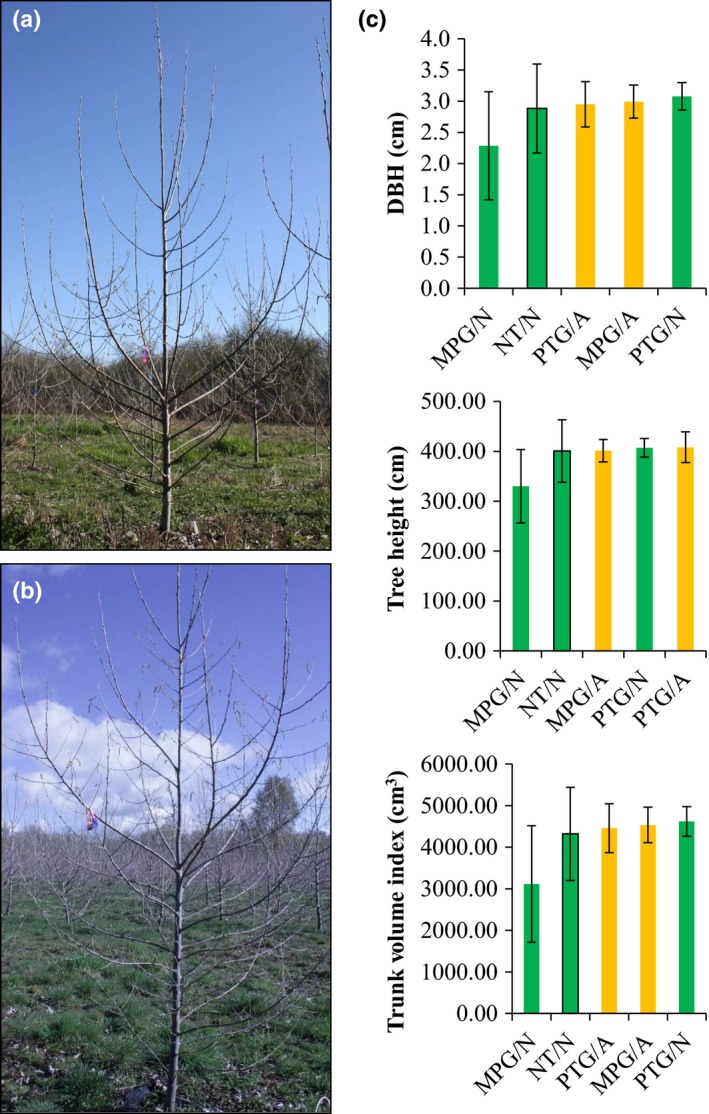
Normal *Populus alba* tree architecture and growth observed in RNA interference events. (a) Tree architecture of nontransformed (NT) control, imaged on 12 March 2014. (b) Tree architecture of the altered event MPG 119, imaged on 14 March 2014. (c) Bar plots of spatially adjusted growth parameters, trunk diameter at breast height (DBH), tree height, and trunk volume index of trees from 2015. PTG/A, altered PTG events; PTG/N, normal PTG events; MPG/A, altered MPG events; MPG/N, normal MPG events; NT/N, nontransgenic control. Bars and error bars represent marginal means and ± SE from the fitted models, respectively.

There were no discernible alterations in tree form in RNAi events compared with NT controls (Fig. 6a,b). Statistical analysis of three growth traits (trunk diameter at breast height, tree height, and trunk volume index) showed no statistically significant changes (*P *<* *0.05) in tree growth among treatments (i.e. altered PTG, normal PTG, altered MPG, normal MPG, and NT control; Fig. 6c, Tables S8, S9). For example, the altered PTG events, on average, were 0.06 mm wider at breast height, 7.16 cm taller and 138.43 cm^3^ larger in volume, compared with the NT control. However, none of these differences were statistically significant (*P *=* *1.00 in these three cases).

**Figure 7:**
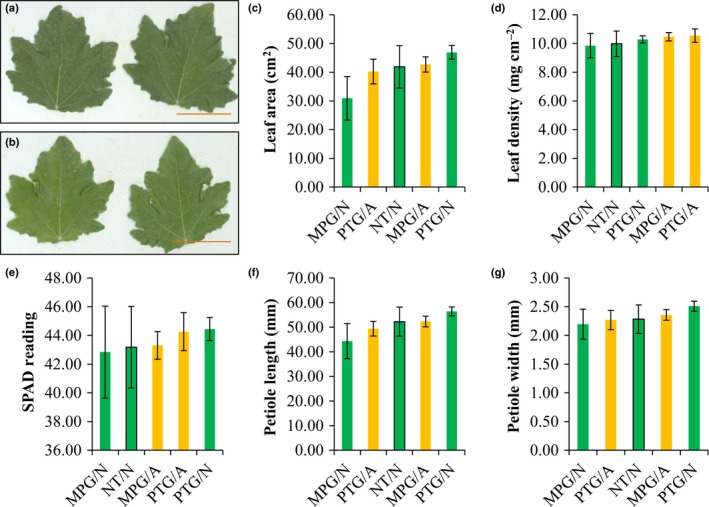
Normal *Populus alba* leaf morphology and growth observed in RNA interference events. (a) Leaf scan of nontransformed (NT) control. (b) Leaf scan of the altered event MPG 119. Images (a) and (b) taken on 21 July 2015. Bars, 5 cm. (c–g) Bar plots of leaf parameters, leaf area (c), leaf density (d), total leaf Chl indicated by SPAD meter reading (e), petiole length (f), and petiole width (g) of leaves collected in summer 2015. PTG/A, altered PTG events; PTG/N, normal PTG events; MPG/A, altered MPG events; MPG/N, normal MPG events; NT/N, nontransgenic control. Bars and error bars represent marginal means and ± SE from the fitted models, respectively.

Similarly, we observed very similar leaf morphology in both RNAi events and NT controls (Fig. 7a,b). For each of the five leaf traits – leaf area, leaf density, Chl content (indicated by SPAD meter reading), petiole length and petiole width – model‐based estimated marginal means of different biological groups were similar (Fig. 7c–g; Table S8). Pairwise comparisons of the estimated marginal means gave *P‐*values higher than 0.29 (Table S9), suggesting no statistically significant differences in leaf traits among altered events, normal events, and NT controls.

## Discussion

The purpose of this study was to use RNAi to suppress the expression of *AG* genes in poplar clone 6K10 (*P. alba*) to illuminate their function in poplar floral and vegetative development, and to hopefully impair fertility as a means of biocontainment. When the RNAi constructs were created, the two poplar *AG* co‐orthologues had been isolated from *P. trichocarpa* (Brunner *et al*., 2000) and served as the sequence resource for vector construction. We found that RNAi suppression of the two *PaAG* genes led to ‘carpel‐inside‐carpel’ phenotypes in 17 out of 24 flowering RNAi events. Despite *PaAG2* sharing high sequence similarity (98.19%) to the *PtAG2* fragment used in the RNAi constructs, we observed stronger suppression in *PaAG1*, which showed 90.7% sequence identity in the region of the RNAi constructs. The two genes are on different chromosomes and have different expression levels (Brunner *et al*., 2000), so perhaps these factors affected susceptibility to RNAi (Samarasinghe *et al*., 2011). Although we did not observe complete conversions of carpels into perianth‐like organs, this is consistent with previous work in *Arabidopsis*. Antisense‐mediated downregulation of *AG* showed that *AG*'s roles in floral organ identity and floral determinacy are separable. Alterations in floral organ identity require a greater reduction in *AG* transcripts than loss of determinacy does (Mizukami & Ma, 1995). We also observed sterile anther‐like organs in 10 out of the 17 altered events (Tables S2, S3). We did not observe detectable or significant changes (*P *<* *0.05) in the architecture of the whole catkins, leaf morphology, tree form, or vegetative growth. These observations demonstrate strong conservation of the roles of *AG* in controlling floral organ determinacy and reproductive organ differentiation in *Populus*.

Although dioecious, *Populu*s retains the ability to produce bisexual flowers and flowers of the alternate sex (Brunner, 2010). Mapping of sex determination regions has not implicated *AG* or other floral homeotic genes as sex determination loci (Kersten *et al*., 2014; Geraldes *et al*., 2015). However, regulation of homeotic gene expression could be a downstream outcome of a sex‐determining gene's activity, and sporadic alterations in their expression by environmental conditions or other factors could bypass the sex determination mechanism. In *Populus*, sex determination occurs before the initiation of reproductive organ primordia. Changes in the expression domains of floral homeotic genes have been linked to flower evolution and diversity, with the relative amounts of AG and B‐class MADS domain proteins proposed to determine the proportions of stamens and carpels (Soltis *et al*., 2007; Liu & Mara, 2010).

*AG* specifies both stamen and carpel identity, but according to the floral quartet model, two AG proteins are needed for the carpel identity quartet, whereas only one is needed for the stamen identity complex that includes two B‐class MADS domain proteins (Theißen *et al*., 2016). *PTD*, a poplar orthologue of the B‐class gene *DEFICIENS* is expressed throughout male and female floral meristems before initiation of floral organ development (Sheppard *et al*., 2000). Whereas *PTD* expression is maintained in stamen primordia, transcripts are excluded from initiating carpel primorida but are present in the space between the carpels and the perianth cup. Moreover, stamens developed around the central indeterminate carpeloid structure in the poplar transgenics (Fig. 2j,k). Perhaps a reduced level of *AG* in female *Populus* allowed the normally repressed male program to manifest in this location by promoting the formation of the stamen‐specifying quartet. Interestingly, downregulation of poplar *LEAFY* (*LFY*) orthologue induced sterility in female clones, but induced bisexual and female flowers in a male clone (Klocko *et al*., 2016b, 2018). Although *LFY* directly activates both *AG* and B‐class genes, *AG* expression is detectable in *lfy* mutants and abnormal carpels develop, but stamens are absent (Siriwardana & Lamb, 2012). Similarly, downregulation of poplar *LFY* could have led to a greater reduction in B‐class protein abundance than in AG amounts, favoring the carpel‐specifying quartet. Considered together, these results suggest that changes in the relative timing, pattern, and level of *AG* and B‐class gene expression could have caused gender alterations and bisexuality in *Populus*.

We observed variation in RNAi‐induced floral morphology among events, within events, and within trees. Because transgenic events can differ from each other in the chromosomal location, structure, chromatin state, and copy number of transgenes (Gelvin, 2003), they will differ in degree and pattern of transgene expression, and consequently will cause variation in target gene suppression and phenotype (Kuhlemeier *et al*., 1987). Transgene expression and associated phenotypes are also known to vary in response to environment and plant developmental stage; thus, it is not surprising that floral structure varied within and among branches, especially in events that had only moderate gene suppression. Despite the phenotypic variations discussed herein, the seedless phenotype was stable within events where the suppression of *PaAG*s and *PaSTK*s was strongest.

In addition to *AG* suppression, we also detected suppression in two *PaSTK* genes – co‐orthologues of *STK* (Klocko *et al*., 2016a), both of which contained two instances of 11–14 contiguous nucleotides that are identical to the *PtAG2*‐derived RNAi hairpin. We did not observe reductions in *Potri.001G254300* expression, which shared only a single instance of eight contiguous identical nucleotides with the RNAi hairpin. These results agree with previous findings that silencing can occur when the nontargeted genes contain as few as 11 contiguous nucleotides of identity to the small interfering RNA (Jackson *et al*., 2003).

The off‐target suppression of two *PaSTK* genes in altered RNAi events was strongly correlated with the seedlessness phenotype (Fig. 5b). Since the cottony seed hair in *Populus* has been found to originate from funiculus – a structure in the ovary that attaches an ovule to the ovary wall (Ye *et al*., 2014) – the absence of seed hair in seedless events (Fig. 4c,d) observed in this study could be an indicator of disrupted ovule and seed development. These results also indicate that the role of *STK* in ovule development is functionally conserved in *Populus* catkins. However, because we did not suppress the two genes independently in this experiment, it is also possible that *PaSTK*s and *PaAG*s play a joint role in regulating ovule development, and it is their combined suppression that caused the seedless phenotype.

Recent studies have suggested a possible role of the *AG* gene in tissue senescence. Deng *et al*. (2011) found that heterologous expression of *NtAG* (an *AG* homologue from *Narcissus tazetta*) in *Arabidopsis* often produced yellow leaves. Compared with wild‐type plants, these transgenics had higher malondialdehyde content and increased expression level of *SENESCENCE ASSOCIATED GENE 12*, both markers of leaf senescence. Furthermore, Jibran *et al*. (2017) reported that flowers of *ag* mutants showed delayed sepal senescence and abscission in *Arabidopsis*, which could be rescued by spraying jasmonate on plants. Contrary to these findings that low *AG* expression retarded or prevented senescence, we noticed accelerated flower senescence in several altered RNAi events (e.g. Fig. S3b). However, we did not observe early leaf senescence in any RNAi events. Our results may be due to a role of *AG* in the flower senescence pathway – partially agreeing with the study done by Jibran *et al*. (2017). Alternatively, the lack of developing ovules, and the presumed hormone‐driven sink function as seeds develop (Bennett *et al*., 2011), would be lacking in sterile catkins. This might also explain their early senescence. A possible role for *AG* in regulating capsule senescence in *Populus*, whether direct or indirect, remains to be tested.

We originally thought that the early floral bud flush in altered RNAi events was due to co‐suppression of dormancy‐associated MADS‐box (DAM) genes (reviewed by Horvath, 2015), which share some sequence identity with the *PaAG* genes. However, our blastn‐based off‐target screen did not hit any DAM genes (Table S7). Additionally, no early vegetative bud flush was observed. Therefore, it is unlikely that our RNAi constructs targeted any DAM genes. The early floral bud flush that we observed might be simply due to the layered, enlarged carpels pushing apart the bud scales prematurely.

MARs have been widely studied as tools for elevating and stabilizing gene expression in plants (e.g. Han *et al*., 1997; Ülker *et al*., 2002; Van der Geest *et al*., 2003; Butaye *et al*., 2004; Abranches *et al*., 2005; Dietz‐Pfeilstetter *et al*., 2016). However, apart from a study where MAR elements elevated the rate of virus‐induced gene silencing (discussed in the following), to our knowledge its value for elevating RNAi efficiency within plants had not been previously addressed. In our study, although the two constructs tested produced similar levels of gene suppression when RNAi was activated, the inclusion of tobacco RB7 MARs in construct MPG led to a more than three‐fold increase in the frequency of RNAi suppression compared with the MAR‐free construct PTG (Table 1). This result provides what is, to our knowledge, the first experimental evidence that flanking‐MARs can elevate RNAi efficiency in plants (Hirai & Kodama, 2008).

Levin *et al*. (2005) evaluated the effect of tobacco RB7 MARs on host‐induced gene silencing. They transformed a virus gene that encodes tomato spotted wilt virus (TSWV) nucleocapsid protein into tobacco with and without flanking MARs. They found that the percentage of plants resistant to tomato spotted wilt virus increased 1.5‐fold in the MAR‐transformed population, and the frequency of loss of resistance in the third generation was four‐fold lower in resistant MAR lines than that in resistant non‐MAR lines. The effect of tobacco RB7 MARs on RNAi in poplar was previously examined by Li *et al*. (2008b). Four RNAi constructs, with or without flanking MARs, were compared for their efficiency in suppressing the herbicide‐resistance gene *BAR* in field‐grown trees. Here, the presence of MARs did not show a discernible influence on the efficiency or stability of RNAi‐induced *BAR* suppression. This lack of effect might be due to the little influence of MARs on RNAi transgene expression level that was reported in the study.

There are widespread concerns over gene flow and dispersal of genetically engineered and invasive exotic plants (Brunner *et al*., 2007; Vining *et al*., 2012; Strauss *et al*., 2017). The conserved function of *AG* and *STK* in *Populus*, as confirmed by our study, could provide an important tool for genetic containment in *Populus*, and likely in many other plant taxa. By prevention of seed production (and we predict also pollen production) through their strong suppression or mutation, it would provide much stronger genetic containment than male sterility alone (the latter has been demonstrated through tapetal ablation in other *Populus* tree species; Zhang *et al*., 2012; Elorriaga *et al*., 2014). Because many tree and shrub species are vegetatively propagated by the forest or horticulture industries, fully sterile RNAi‐*AG* trees could still be amplified and planted.

In addition to RNAi technology, whose efficacy has been demonstrated here and by others (e.g. Li *et al*., 2008b), the use of site‐directed mutagenesis methods such as CRISPR/Cas9 can be highly effective for generating loss‐of‐function mutations, including in *Populus* (Fan *et al*., 2015; Zhou *et al*., 2015; Elorriaga *et al*., 2018). This could lead to much stronger and more stable phenotypes than using RNAi. Our results suggest that such a strategy might be highly effective and free of pleiotropic vegetative effects when the two *AG* and/or the two *STK* orthologues are mutated.

## Author contribution

SHS oversaw the work and led or co‐led, along with ALK and AMB, the several grant proposals that funded the project. HL, ALK, AMB, CM, ACM and XA conducted the experiments; HL and GTH analyzed the data; HL and SHS wrote the manuscript.

## Supporting information

Please note: Wiley Blackwell are not responsible for the content or functionality of any Supporting Information supplied by the authors. Any queries (other than missing material) should be directed to the *New Phytologist* Central Office.

**Fig. S1** Alignment of *PtAG2*,* PaAG1* and *PaAG2* cDNA sequences.
**Fig. S2** Morphological variation was commonly observed among events.
**Fig. S3** Floral morphology was stable over multiple years.
**Fig. S4** Floral bud opening in field.
**Fig. S5** Relative transcript level of *Potri.001G254300* in developing floral buds by event, treatment and construct.

**Table S1** Primers used for cDNA sequencing or for measuring gene expression via qRT‐PCR.
**Table S2** Tree survival, flowering and floral morphology of PTG events in 2018.
**Table S3** Tree survival, flowering and floral morphology of MPG events in 2018.
**Table S4** Seed germination of constructs and events.
**Table S5** Relative gene expression of five genes in transgenic events and NT control trees.
**Table S6** Pearson correlation coefficient between gene expression.
**Table S7** Potential off‐target genes identified by Blastn.
**Table S8** Marginal means and SEs for growth and leaf morphology of five treatment combinations of construct (PTG, MPG, and NT) and floral morphology.
**Table S9** *P*‐values associated with differences among five treatment combinations of construct (PTG, MPG and NT) and floral morphology.
