# Supplementary material for: RNA interference suppression of AGAMOUS and SEEDSTICK alters floral organ identity and impairs floral organ determinacy, ovule differentiation, and seed‐hair development in Populus
Source: New Phytol. 2019 Jan 19;222(2):923–37. doi: 10.1111/nph.15648 (PMC6590139; doi:10.1111/nph.15648)
Supplement: Supplementary file 1 — Fig. S1 Alignment of PtAG2, PaAG1 and PaAG2 cDNA sequences. Fig. S2 Morphological variation was commonly observed among events. Fig. S3 Floral morphology was stable over multiple years. Fig. S4 Floral bud opening in field. Fig. S5 Relative transcript level of Potri.001G254300 in developing floral buds by event, treatment and construct. [file NPH-222-923-s001.pdf]

```
PtAG2     AGCTAGGCAGCAGCTATGGCATACCAAAATGAACCCCAAGAGAGCTCTCCCCTGAGGAAG 60
PaAG1     AGCTAGACTGCAGCTATGGAATATCAAAATGAATCCCTTGAGAGCTCCCCCCTGAGGAAG 60
PaAG2     AGCTAGGCAGCAGCCATGGCATACCAAAATGMATCCCAAGAGAGCTCTCCCCTGAGGAAG 60
          ****** * ***** **** *** ******* * ***  ******** ************

PtAG2     CTGGGGAGGGGAAAGGTGGAGATCAAGCGGATCGAGAACACCACCAATCGCCAAGTCACT 120
PaAG1     CTGGGAAGGGGAAAGGTGGAGATCAAGCGGATCGAGAACACCACCAATCGCCAAGTCACT 120
PaAG2     CTGGGGAGGGGAAAGGTGGAGATCAAGCGGATCGAGAACACCACCAATCGCCAAGTTACT 120
          ***** ************************************************** ***

PtAG2     TTCTGCAAAAGGCGGAATGGTTTGCTCAAGAAAGCCTATGAATTATCTGTTCTTTGCGAT 180
PaAG1     TTCTGCAAAAGGCGCAGTGGTTTGCTCAAGAAAGCCTACGAATTATCTGTTCTTTGCGAT 180
PaAG2     TTCTGCAAAAGGCGGAATGGTTTGCTCAAGAAAGCCTATGAATTATCTGTTCTTTGCGAT 180
          ************** * ********************* *********************

PtAG2     GCTGAGGTTGCACTCATCGTCTTCTCCAGCCGTGGACGCCTTTATGAGTACTCTAACAAT 240
PaAG1     GCTGAGGTTGCACTCATCGTCTTCTCTACCCGCGGTCGCCTTTATGAGTACTCTAACGAT 240
PaAG2     GCTGAGGTTGCACTCATCGTCTTCTCCAGCCGTGGACGCCTTTATGAGTACTCTAACAAT 240
          ************************** * *** ** ********************* **

PtAG2     AGTGTCAAATCTACAATTGAAAGGTACAAAAAGGCATGTGCAGATTCTTCCAACAACGGG 300
PaAG1     AGTGTCAAATCAACAATTGAGAGGTACAAAAAGGCATCTGCAGATTCTTCAAACACTGGG 300
PaAG2     AGTGTCAAATCTACAATTGAAAGGTACAAAAAGGCATGTGCAGATTCTTCCAACAACGGG 300
          *********** ******** **************** ************ ****  ***

PtAG2     TCAGTTTCTGAAGCCAATGCTCAGTTTTATCAGCAAGAAGCTGCCAAGCTGCGCTCGCAA 360
PaAG1     TCTGTTTCTGAAGCCAATGCTCAGTACTACCAGCAAGAAGCTGCCAAGCTGCGTTCCCAA 360
PaAG2     TCAGTTTCTGAAGCCAATGCTCAGTTCTATCAGCAAGAAGCTGCCAAGCTGCGCTCGCAA 360
          ** **********************  ** *********************** ** ***

PtAG2     ATTGGTAATTTGCAGAATTCAAACAGGAATATGCTGGGTGAATCACTTAGTGCATTGAGT 420
PaAG1     ATTGGTAATTTGCAGAATTCAAACAGGCATATGCTGGGTGAAGCTCTTAGTTCATTGAGT 420
PaAG2     ATTGGTAATTTGCAGAATTCAAACAGGAACATGCTGGGTGAATCACTTAGTGCATTGAGC 420
          *************************** * ************ * ****** *******

PtAG2     GTGAAGGAACTTAAGAGCTTGGAGATAAAACTTGAGAAAGGAATTGGTAGAATTCGTTCG 480
PaAG1     GTGAAGGAACTTAAGAGTTTGGAAATACGACTTGAGAAAGGAATAAGCAGAATTCGTTCC 480
PaAG2     GTGAAGGAACTTAAGAGCTTGGAGATAAAACTTGAGAAAGGAATTGGTAGAATTCGTTCG 480
          ***************** ***** ***  ***************  * ***********

PtAG2     AAAAAGAATGAGCTGTTGTTTGCTGAAATTGAGTATATGCAGAAGAGGGAGATTGACTTG 540
PaAG1     AAAAAGAATGAGCTGTTGTTTGCAGAAATCGAGTATATGCAGAAGAGGGAGGTTGACTTG 540
PaAG2     AAAAAGAATGAGCTGTTGTTTGCTGAAATAGAGTATATGCAGAAGAGGGAGATTGACTTG 540
          *********************** ***** ********************* ********

PtAG2     CACAACAATAACCAGCTTCTCCGAGCAAAGATTGCAGAGAATGAAAGAAAGCGACAGCAC 600
PaAG1     CACAACAACAACCAGCTTCTCCGAGCAAAGATTTCAGAGAATGAAAGAAAGCGACAGAGC 600
PaAG2     CACACCAATAACCAGCTTCTCCGAGCAAAGATTGCAGAGAACGAAAGAAAGCGACAGCAC 600
          **** *** ************************ ******* ***************  *

PtAG2     ATGAATTTGATGCCGGGAGGTGTCAACTTCGAGATCATGCAGTCTCAACCATTTGACTCT 660
PaAG1     ATGAATTTGATGCCAGGAGGAGCAGACTTTGAGATCGTGCAGTCTCAACCATACGACTCT 660
PaAG2     ATGAATTTGATGCCAGGAGGTGTCAACTTCGAGATCATGCAGTCTCAACCATTTGACTCT 660
          ************** ***** *   **** ****** ***************  ******

PtAG2     CGGAACTATTCTCAAGTTAATGGATTGCCGCCTGCCAATCATTACCCTCATGAAGACCAG 720
PaAG1     CGCAACTATTCTCAAGTGAATGGAATACCGCCTGCAAGTCATTACTCACATCAAGATCAG 720
PaAG2     CGGAACTATTCTCAAGTTAATGGATTGCCGCCTGCCAATCATTACCCTCACGAAGACCAG 720
          ** ************** ****** * ******** * ******* * **  **** ***

PtAG2     CTCT----TCAGTTAGTTTAAAAAGCACCAAGTGCAGCAACTCCTCGCATTTCC       770
PaAG1     ATGGCCCTTCAGTTAGTTTAATAATCTCCAAGGGCAGCAGTTTCTCGCATTTCC       774
PaAG2     CTCT----TCAGTTAGTTTTAAAAGCACCAAGTGCAGCAACTCCTCGCGTTCCA       770
           *      *********** * ** * ***** ******  * ***** ** *
```

**Fig. S1** Alignment of *PtAG2*, *PaAG1* and *PaAG2* cDNA sequences. Asterisks indicate conserved residues. M indicates A and C. The line under the alignment indicates the portion of the cDNA sequence used to make the RNAi hairpin molecule. Numbers on right indicate base pairs.

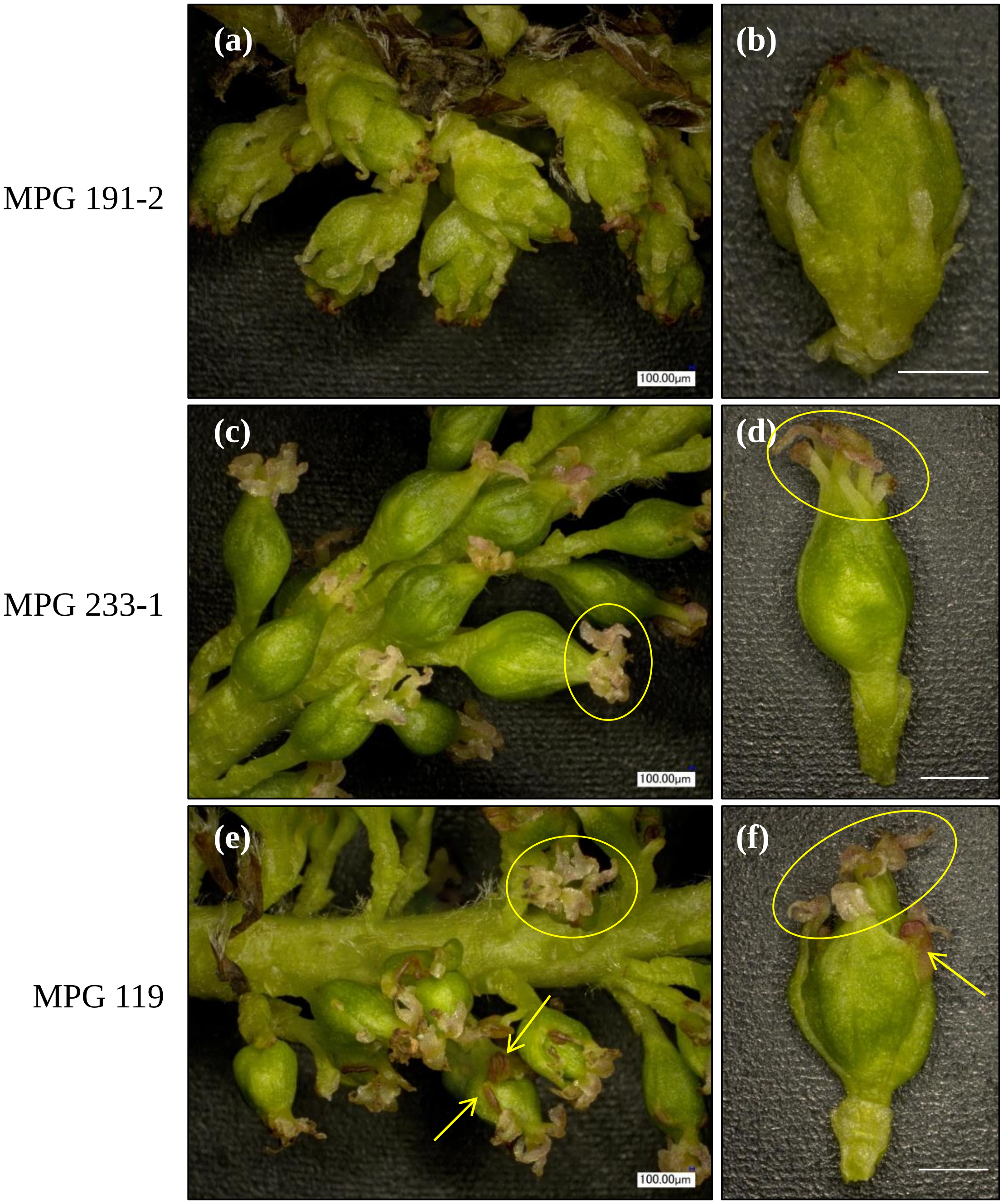


**Fig. S2** Morphological variation was commonly observed among events. (a – b) Catkins and individual flowers from event MPG 191-2. (c – d) Catkins and individual flowers from event MPG 233-1. (e – f) Catkins and individual flowers from event MPG 119. Layered carpels and the consequently increased number of stigmas indicated are by circles; underdeveloped anther-like organs are indicated by arrows. White scale bars in images b, d and f indicate 1 mm. (All images taken on March 21, 2016.)

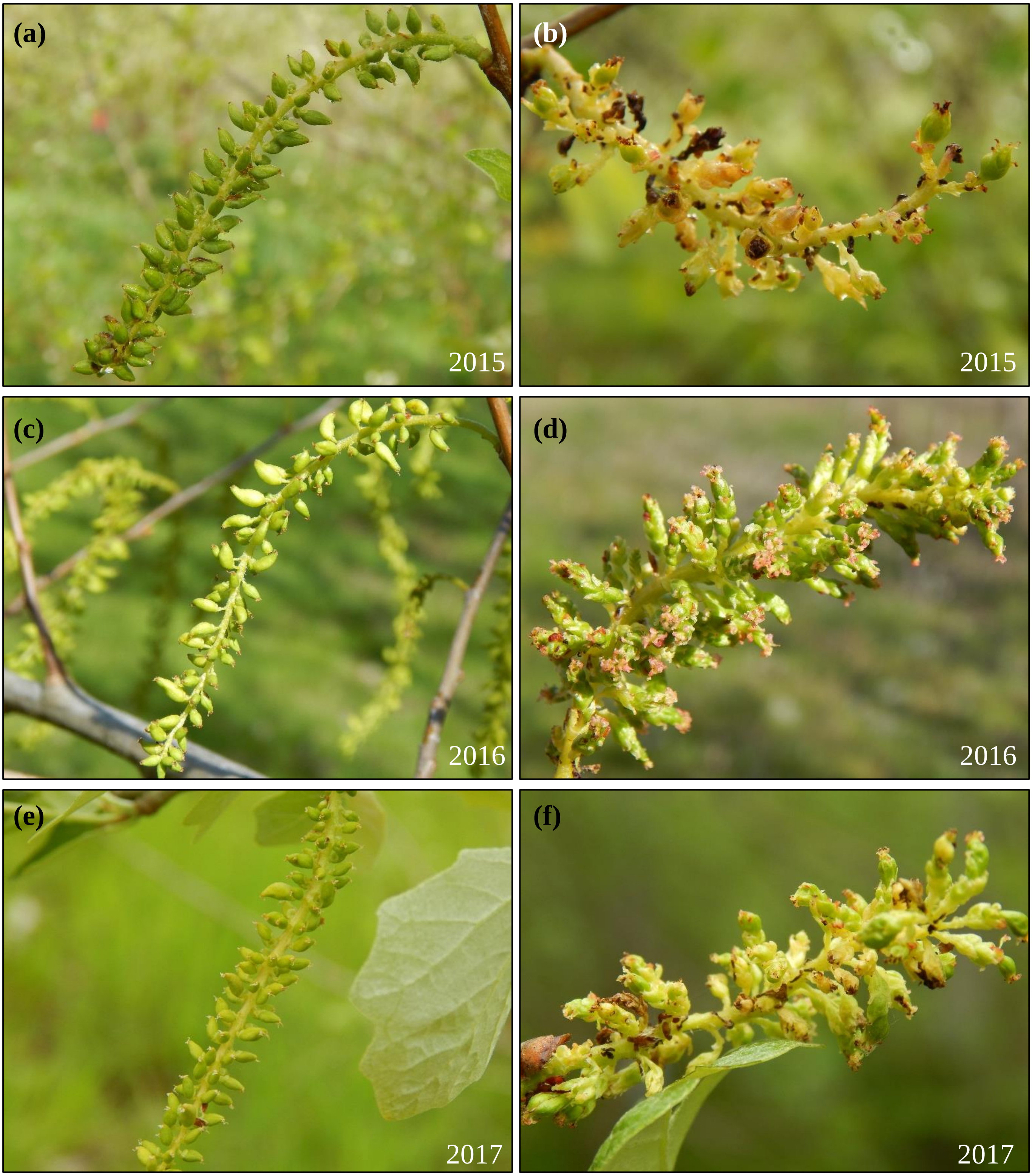


**Fig. S3** Floral morphology was stable over multiple years. (a, c, and e) Nearly mature catkins from NT control. (b, d and f) Catkins from MPG event 165-1. (Images a and b taken on April 2, 2015, c and d on March 17, 2016 and e and f on April 30, 2017.)

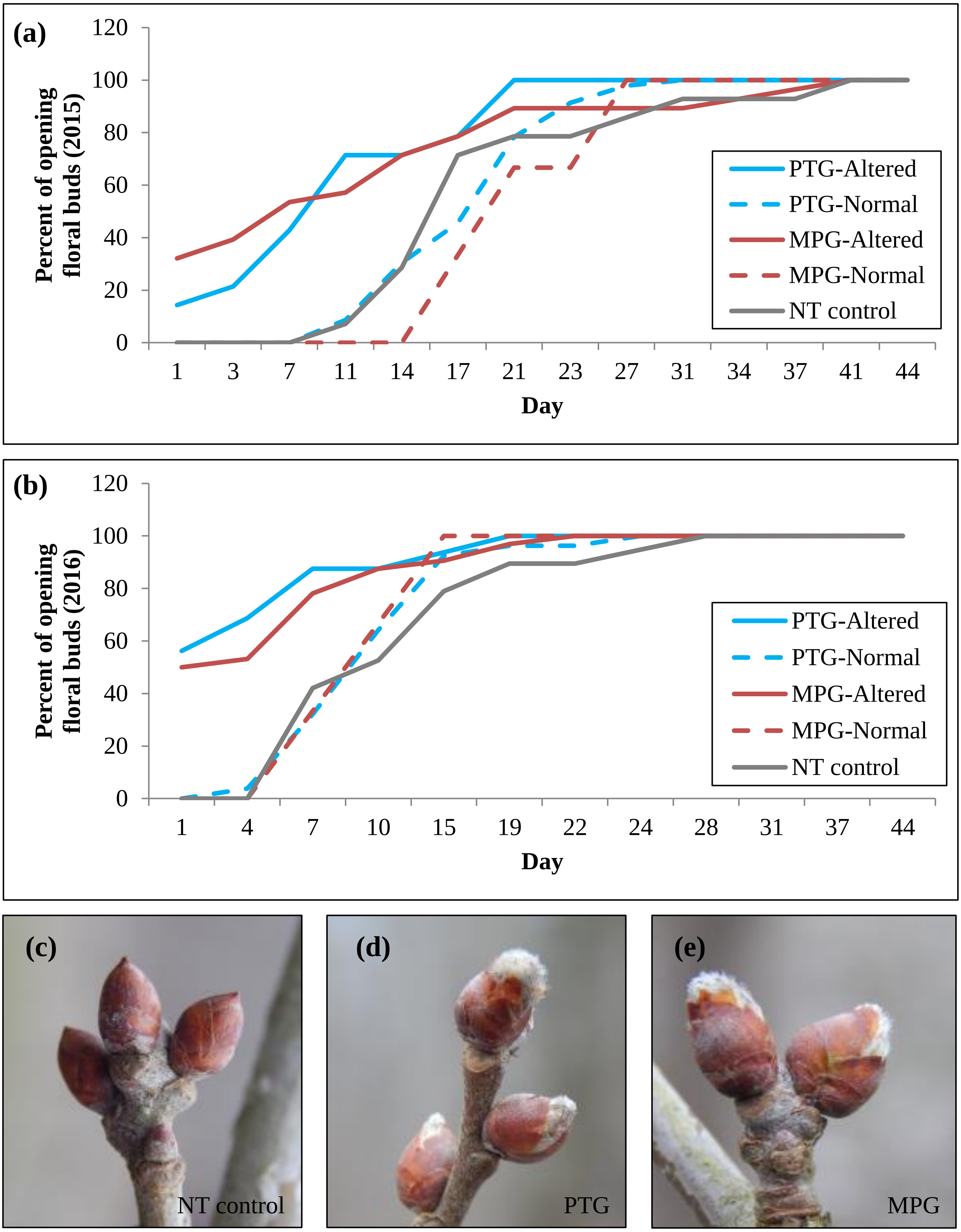


**Fig. S4** Floral bud opening in field. (a) 2015 data, collected beginning January 28 (day 1). (b) 2016 data, collected beginning February 2 (day 1). (c) Closed floral buds on NT control 8. (d) Early-opening floral buds on the altered event PTG 119. (e) Early-opening floral buds on the altered event MPG 165-1. (Images c – e taken on February 1, 2017.)

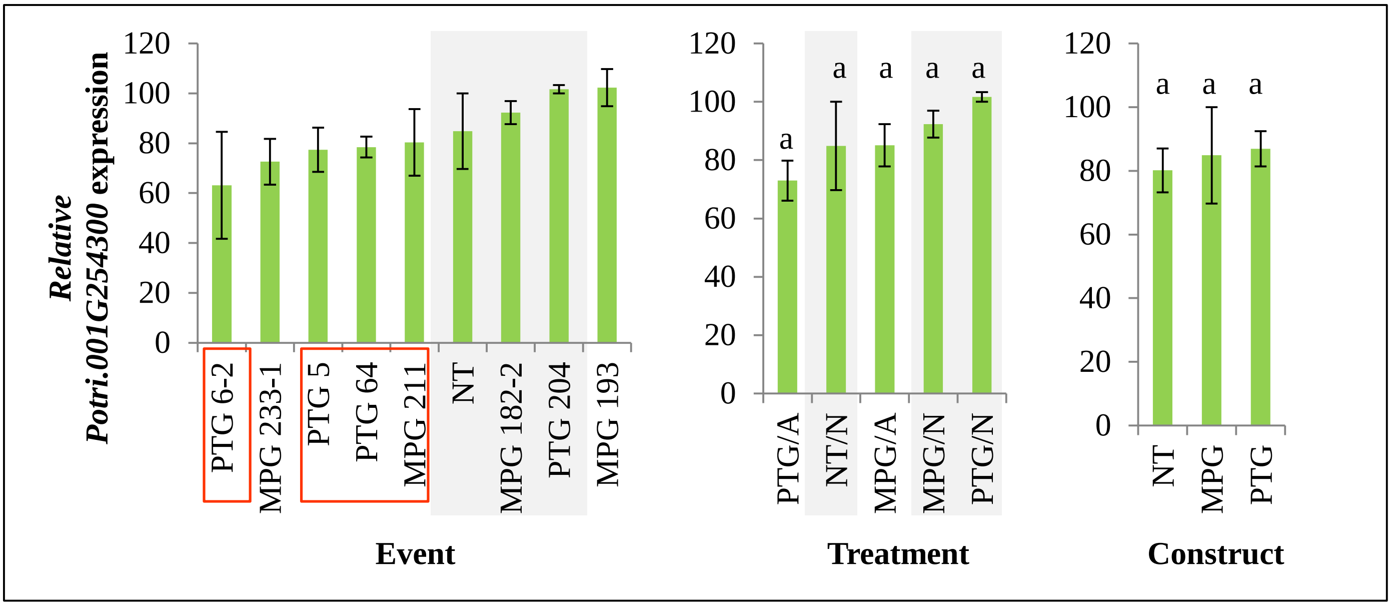


**Fig. S5** Relative transcript level of *Potri.001G254300* in developing floral buds by event, treatment and construct. Error bars represent standard errors of means. Letters denote significant differences ($P < 0.05$). Normal events and NT control are highlighted in grey. Seedless events are indicated by orange boxes. The five treatments: PTG/A = altered PTG events, PTG/N = normal PTG events, MPG/A = altered MPG events, MPG/N = normal MPG events, NT/N = non-transgenic control.
