# Supplementary material for: RNA interference suppression of AGAMOUS and SEEDSTICK alters floral organ identity and impairs floral organ determinacy, ovule differentiation, and seed‐hair development in Populus
Source: New Phytol. 2019 Jan 19;222(2):923–37. doi: 10.1111/nph.15648 (PMC6590139; doi:10.1111/nph.15648)
Supplement: Supplementary file 2 — Table S1 Primers used for cDNA sequencing or for measuring gene expression via qRT‐PCR. Table S2 Tree survival, flowering and floral morphology of PTG events in 2018. Table S3 Tree survival, flowering and floral morphology of MPG events in 2018. Table S4 Seed germination of constructs and events. Table S5 Relative gene expression of five genes in transgenic events and NT control trees. Table S6 Pearson correlation coefficient between gene expression. Table S7 Potential off‐target genes identified by Blastn. Table S8 Marginal means and SEs for growth and leaf morphology of five treatment combinations of construct (PTG, MPG, and NT) and floral morphology. Table S9 P‐values associated with differences among five treatment combinations of construct (PTG, MPG and NT) and floral morphology. [file NPH-222-923-s002.pdf]

Table S1 Primers used for cDNA sequencing or for measuring gene expression via qRT-PCR. Amplification efficiency and correlation coefficient R^2 from standard curve included for each qRT-PCR primer pair. N/A indicates that amplification efficiency and correlation with control template concentration (R^2) were not evaluated.

**Gene ID/Name**	**Forward Primer Sequence (5' - 3')**	**Reverse Primer Sequence (5' - 3')**	**Purpose**	**Efficiency (%)**	**R^2**
Potri.004G064300/*AG1*	AAG GAA AAG ATC CTC ACT TTC TCT ACA C	ATA GTT AAT ACA TAG AGG AAG AGT ACT AGG C	Sequencing	N/A	N/A
Potri.011G075800/*AG2*	GAG CAA AAG ATC CTT ACT TTC TCT CC	GCT TTT GCG CTC AGT GAA ATA GT	Sequencing	N/A	N/A
Potri.009G01860/*EF1-beta*	AAC CTG GTC GTG ATT TCC CT	ATC ACC AGC AGC CTC CTT G	qRT-PCR	100.492	0.996
Potri.018G10730/*eIF5A*	TCG TTC CTT CAT CTC ACA ACT GT	AGA CTC ACA AAG CCA TCT TCA GA	qRT-PCR	94.112	0.978
Potri.004G064300/*AG1*	CAG TGG TTT GCT CAA GAA AGC CTAC	ATT GGC TTC AGA AAC AGA CCC AG	qRT-PCR	108.040	0.970
Potri.011G075800/*AG2*	GAA TGG TTT GCT CAA GAA AGC CTA T	CTT CAG AAA CTG ACC CGT TGT TG	qRT-PCR	101.081	0.982
Potri.013G104900/*STK-2*	CGA AAG AGG CAT GAC TAG AAT CAG	GGT GAC CAT GTT GGC TTG TTG	qRT-PCR	103.140	0.980
Potri.019G077200/*STK-1*	TGA GAG AGG CAT TAC TAG AAT CAG G	AGT CAC CAT GTT GGC TTG TTG A	qRT-PCR	109.449	0.958
Potri.001G254300*	GTT GGT CAA GCA TTC CCT GTC TA	TAT TAA ACA AGC AAT CTG GCT CAT CA	qRT-PCR	95.983	0.993

* No gene name available; the functional annotation is nucleosome-remodeling factor subunit BPTF.

Table S2 Tree survival, flowering and floral morphology of PTG events in 2018. Altered events are highlighted in grey. "Altered" indicates the flowers are abnormal, with either a "carpel-inside-carpel" phenotype (layered) or the presence of well-developed or underdeveloped anther-looking organs (anthers).

Event	No. of Ramets Planted in 2011	Survival (No. and Percent of Ramets)	Flowering (No. and Percent of Ramets)	Floral Morphology
5	4	4 (100.0%)	2 (50.0%)	Altered (layered)
6-2	4	4 (100.0%)	4 (100.0%)	Altered (layered, anthers)
8	4	4 (100.0%)	3 (75.0%)	Normal
11	4	2 (50.0%)	1 (50.0%)	Altered (layered)
48	4	4 (100.0%)	4 (100.0%)	Normal
64	4	4 (100.0%)	4 (100.0%)	Altered (layered, anthers)
72	4	4 (100.0%)	4 (100.0%)	Normal
81	3	3 (100.0%)	3 (100.0%)	Normal
84	4	4 (100.0%)	4 (100.0%)	Normal
87	4	4 (100.0%)	3 (75.0%)	Normal
90-1	4	4 (100.0%)	4 (100.0%)	Normal
93-1	4	4 (100.0%)	4 (100.0%)	Altered (layered, anthers)
116-2	4	4 (100.0%)	4 (100.0%)	Normal
174	4	4 (100.0%)	4 (100.0%)	Normal
178	4	4 (100.0%)	4 (100.0%)	Normal
179-2	4	4 (100.0%)	4 (100.0%)	Normal
195-1	8	8 (100.0%)	8 (100.0%)	Normal
198	4	3 (75.0%)	3 (100.0%)	Normal
199	4	4 (100.0%)	4 (100.0%)	Altered (layered, anthers)
204	4	4 (100.0%)	4 (100.0%)	Normal
207	4	4 (100.0%)	4 (100.0%)	Normal
211	2	1 (50.0%)	1 (100.0%)	Normal
**Total 22**	**89**	**85**	**80**	**6 altered events; 16 normal events**

Table S3 Tree survival, flowering and floral morphology of MPG events in 2018. Altered events are highlighted in grey. "Altered" indicates the flowers are abnormal, with either a "carpel-inside-carpel" phenotype (layered) or the presence of well-developed or underdeveloped anther-looking organs (anthers). Due to the absence of flowers, floral morphology was unavailable (NF) for event 91-2.

Event ID	No. of Ramets Planted in 2011	Survival (No. and Percent of Ramets	Flowering (No. and Percent of Ramets)	Floral Morphology
28-1	4	4 (100.0%)	4 (100.0%)	Altered (layered)
91-2	1	1 (100.0%)	0 (0.0%)	NF
116-1	4	4 (100.0%)	4 (100.0%)	Altered (layered, anthers)
119	4	4 (100.0%)	4 (100.0%)	Altered (layered, anthers)
148-3	4	4 (100.0%)	4 (100.0%)	Altered (layered)
165-1	4	4 (100.0%)	4 (100.0%)	Altered (layered, anthers)
182-2	4	4 (100.0%)	4 (100.0%)	Normal
191-2	3	3 (100.0%)	2 (66.7%)	Altered (layered)
193	4	4 (100.0%)	4 (100.0%)	Altered (layered, anthers)
194-2	4	4 (100.0%)	2 (50.0%)	Altered (layered)
203-2	4	4 (100.0%)	4 (100.0%)	Altered (layered, anthers)
211	4	4 (100.0%)	3 (75.0%)	Altered (layered)
233-1	4	4 (100.0%)	4 (100.0%)	Altered (layered, anthers)
**Total 13**	**48**	**48**	**43**	**11 altered events;<br>1 normal event;<br>1 non-flowering event**

Table S4 Seed germination of constructs and events. PTG, RNAi construct without MARs; MPG, RNAi construct with flanking MARs; NT, non-transgenic control. Infertile events are highlighted in grey. Asterisks identify events that had a normal floral morphology. No event ID was designated (indicated by "ND") for NT control.

Construct	Event	No. of Trees (ramets)	No. of Carpels Dissected	No. of Seeds Planted	No. of Seeds Germinated	Seeds per Carpel	Germinated seeds per Carpel
PTG	5	2	35	0	0	0.00	0.00
	6-2	2	15	0	0	0.00	0.00
	64	1	15	0	0	0.00	0.00
	199	4	42	16	8	0.38	0.19
	93-1	2	40	39	11	0.98	0.28
	207*	2	37	52	34	1.40	0.92
MPG	191-2	2	30	0	0	0.00	0.00
	193	2	30	0	0	0.00	0.00
	194-2	2	30	0	0	0.00	0.00
	211	2	30	0	0	0.00	0.00
	116-1	2	30	3	0	0.10	0.00
	28-1	1	30	3	0	0.10	0.00
	203-2	2	50	5	0	0.10	0.00
	165-1	2	50	5	2	0.10	0.04
	233-1	4	97	43	17	0.44	0.18
	148-3	2	40	16	10	0.40	0.25
	119	1	40	19	10	0.48	0.25
	182-2*	2	33	55	38	1.67	1.15
NT	ND	2	59	82	70	1.40	1.20

Table S5 Relative gene expression of five genes in transgenic events and NT control trees. Events with altered floral morphology are highlighted in grey. Parentheses indicate standard errors (SEs). Asterisks indicate statistically significant differences compared with NT control: ***, $P < 0.001$; **, $P < 0.01$; *, $P < 0.05$. NE indicates that gene expression was not examined, ND = event ID not designated.

Construct	Event	*PaAG1*	*PaAG2*	*PaAGs*	*PaSTK-1*	*PaSTK-2*	*PaSTKs*	*PaAGs* + *PaSTKs*	*Potri.001G254300*
PTG	5	18.98 (±4.85)***	29.61 (±0.43)***	48.59***	37.50 (±1.93)***	40.13 (±1)***	77.63***	126.22***	77.40 (±8.85)
	6-2	17.37 (±0.12)***	21.43 (±5.33)***	38.80***	8.37 (±2.00)***	7.93 (±2.27)***	16.30***	55.10***	63.08 (±21.45)
	11	57.00 (±2.39)***	49.13 (±1.47)***	106.13***	53.41 (±1.62)***	54.50 (±2.1)***	107.91***	214.04***	NE
	64	25.20 (±1.06)***	29.34 (±4.09)***	54.54***	15.20 (±0.38)***	16.05 (±0.87)***	31.25***	85.79***	78.49 (±4.23)
	93-1	28.63 (±2.66)***	35.37 (±1.37)***	64.00***	82.46 (±1.11)	73.03 (±1.99)	155.49	219.49***	NE
	199	23.60 (±1.36)***	31.75 (±7.3)***	55.35***	66.81 (±3.48)**	71.68 (±4.87)	138.49*	193.84***	NE
	204	81.47 (±6.2)	87.20 (±6.43)	168.67	86.36 (±0.57)	82.69 (±0.54)	169.05	337.72	101.68 (±1.68)
MPG	28-1	31.57 (±0.1)***	66.10 (±16.32)**	97.67***	82.26 (±0)	90.71 (±0)	172.97	270.64***	NE
	116-1	32.11 (±0.77)***	56.47 (±1.91)***	88.58***	80.94 (±2.82)	89.35 (±3.67)	170.29	258.87***	NE
	119	43.52 (±0.56)***	75.38 (±0.81)	118.90***	83.90 (±3.88)	85.28 (±5.69)	169.18	288.08***	NE
	148-3	29.53 (±1.83)***	42.93 (±2.88)***	72.46***	72.96 (±6.27)	90.59 (±4.13)	163.55	236.01***	NE
	165-1	32.67 (±3.46)***	47.30 (±19.25)***	79.97***	55.26 (±11.35)***	67.70 (±13.58)*	122.96**	202.93***	NE
	182-2	80.70 (±11.29)	93.45 (±11.06)	174.15	90.98 (±9.12)	81.76 (±6.85)	172.74	346.89	92.35 (±4.62)
	193	31.36 (±1.82)***	56.56 (±1.93)***	87.92***	35.96 (±4.87)***	43.83 (±0.42)***	79.79***	167.71***	102.30 (±7.44)
	203-2	26.00 (±2.82)***	34.58 (±5.04)***	60.58***	67.22 (±3.12)**	65.23 (±0.49)*	132.45**	193.03***	NE
	211	19.33 (±2.55)***	31.66 (±1.07)***	50.99***	18.96 (±1.62)***	26.49 (±2.1)***	45.45***	96.44***	80.41 (±13.36)
	233-1	20.63 (±2.51)***	24.74 (±5.25)***	45.37***	71.55 (±7.34)*	75.90 (±7.27)	147.45	192.82***	72.59 (±9.16)
NT	ND	94.63 (±1.48)	99.99 (±1.62)	194.62	99.17 (±0.83)	95.94 (±4.06)	195.11	389.73	84.88 (±15.12)

Table S6 Pearson correlation coefficient between gene expression. Asterisks indicate statistical significance at event level (n= 18): ***, $P < 0.001$; **, $P < 0.01$; *, $P < 0.05$.

**Gene**	***PaAG1***	***PaAG2***	***PaSTK-1***	***PaSTK-2***
PaAG1	1.00	0.90***	0.60**	0.49*
PaAG2	0.90***	1.00	0.68**	0.63**
PaSTK-1	0.60**	0.68**	1.00	0.97***
PaSTK-2	0.49*	0.63**	0.97***	1.00

Table S7 Potential off-target genes identified by BLASTN. Genes selected for qRT-PCR analysis are highlighted in grey.

Gene ID	Functional Annotation	Contiguous Nucleotides Identical to the 386-bp *PtAG2* cDNA sequence		Expression in female flower (Ave FPKM*)
		Max Length (nts)	No. of instances (≥ 8 nts)	
Potri.013G104900 (*PtSTK2*)	MADS box transcription factor	14	3 (1 × 14 nts, 1× 11 nts, 1× 8 nts)	0.94
Potri.019G077200 (*PtSTK1*)	MADS box transcription factor	14	3 (1 × 14 nts, 1× 11 nts, 1× 8 nts)	1.40
Potri.006G031600	Phosphatidylinositol 4-kinase	8	2 (× 8 nts)	0.18
Potri.001G254300	Nucleosome-remodeling factor subunit	8	1 (× 8 nts)	4.54
Potri.013G043100	Phospholipase A	8	1 (× 8 nts)	2.41
Potri.001G415900	Cell cycle control protein	7	0	1.69
Potri.011G015600	Diphosphate-fructose-6-phosphate 1-phosphotransferase	7	0	6.80
Potri.011G015900	Pyrophosphate-fructose-6-phosphate 1-phosphotransferase	7	0	19.22
Potri.004G003800	Pyrophosphate--fructose-6-phosphate 1-phosphotransferase	7	0	13.90
Potri.005G176600	Transcription initiation factor TFIIH subunit	6	0	0.76
Potri.007G032700	Transcription factor MEIS1 and related HOX domain proteins	6	0	2.70
Potri.010G027000	Nucleoside hydrolase	5	0	0.02
Potri.014G142900	Vesicle protein sorting-associated	4	0	2.18

* Ave FPKM was calculated based on *Populus* GeneAtlas experiments BESC423.ZL female early, BESC443.ZG female receptive and BESC842.ZI female late (Phytozome 12).

Table S8 Marginal means and standard errors (SEs) for growth and leaf morphology of five treatment combinations of construct (PTG, MPG, and NT) and floral morphology (normal = N and altered = A). Statistics were calculated using the statistical models described in the Materials and Methods.

Trait	PTG/A	PTG/N	MPG/A	MPG/N	NT/N
DBH (cm)	2.95 (±0.36)	3.08 (±0.22)	2.99 (±0.27)	2.29 (±0.87)	2.88 (±0.71)
Height (cm)	408.05 (±30.71)	406.77 (±18.57)	401.41 (±22.46)	329.95 (±73.62)	400.89 (±62.58)
Trunk volume index (cm^3)	4457.44 (±587.48)	4617.75 (±354.19)	4535.99 (±428.73)	3114.92 (±1402.2)	4319.01 (±1122.42)
Leaf area (cm^2)	40.26 (±4.29)	46.9 (±2.38)	42.69 (±2.61)	30.94 (±7.59)	41.87 (±7.36)
Leaf density (mg/cm^2)	10.55 (±0.46)	10.28 (±0.25)	10.48 (±0.29)	9.85 (±0.84)	9.98 (±0.89)
SPAD reading	44.26 (±1.33)	44.44 (±0.80)	43.30 (±0.96)	42.84 (±3.20)	43.18 (±2.85)
Petiole length (mm)	49.37 (±2.99)	56.42 (±1.80)	52.33 (±2.16)	44.33 (±7.18)	52.25 (±5.93)
Petiole width (mm)	2.27 (±0.17)	2.51 (±0.09)	2.36 (±0.09)	2.20 (±0.26)	2.28 (±0.25)

Table S9 *P*-values associated with differences among five treatment combinations of construct (PTG, MPG, and NT) and floral morphology (normal = N and altered = A).

Trait	Difference in Marginal Mean (*P*-value)									
	PTG/A - NT/N	PTG/A - PTG/N	PTG/A- MPG/N	MPG/A - PTG/A	MPG/A - NT/N	MPG/A - PTG/N	MPG/A - MPG/N	PTG/N - NT/N	MPG/N - NT/N	PTG/N - MPG/N
DBH (cm)	0.06 (1.00)	-0.13 (1.00)	0.66 (0.95)	-0.04 (1.00)	0.11 (1.00)	-0.08 (1.00)	0.71 (0.93)	0.19 (1.00)	-0.60 (0.98)	0.79 (0.90)
Height (cm)	7.16 (1.00)	1.28 (1.00)	78.10 (0.86)	6.64 (1.00)	0.52 (1.00)	-5.36 (1.00)	71.46 (0.88)	5.88 (1.00)	-70.94 (0.95)	76.82 (0.85)
Trunk volume index (cm^3)	138.43 (1.00)	-160.31 (1.00)	1342.52 (0.90)	-78.55 (1.00)	216.97 (1.00)	-81.76 (1.00)	1421.07 (0.87)	298.74 (1.00)	-1204.09 (0.96)	1502.83 (0.84)
Leaf area (cm^2)	-1.61 (1.00)	-6.64 (0.66)	9.31 (0.82)	-2.44 (0.99)	0.82 (1.00)	-4.21 (0.76)	11.75 (0.59)	5.03 (0.97)	-10.92 (0.84)	15.95 (0.29)
Leaf density (mg/cm^2)	0.57 (0.98)	0.27 (0.99)	0.70 (0.95)	0.07 (1.00)	0.50 (0.98)	0.20 (0.99)	0.63 (0.95)	0.30 (1.00)	-0.13 (1.00)	0.43 (0.99)
SPAD reading	1.08 (1.00)	-0.19 (1.00)	1.42 (0.99)	0.96 (0.98)	0.12 (1.00)	-1.14 (0.89)	0.46 (1.00)	1.27 (0.99)	-0.34 (1.00)	1.60 (0.99)
Petiole length (mm)	-2.88 (0.99)	-7.04 (0.28)	5.04 (0.97)	-2.95 (0.93)	0.08 (1.00)	-4.09 (0.60)	7.99 (0.82)	4.17 (0.96)	-7.92 (0.91)	12.08 (0.49)
Petiole width (mm)	-0.01 (1.00)	-0.24 (0.71)	0.07 (1.00)	-0.09 (0.99)	0.07 (1.00)	-0.15 (0.76)	0.16 (0.98)	0.23 (0.91)	-0.09 (1.00)	0.31 (0.79)
